# Assessing the impact of a food supplement on the nutritional status and body composition of HIV-infected Zambian women on ARVs

**DOI:** 10.1186/1471-2458-11-714

**Published:** 2011-09-21

**Authors:** Rodah M Zulu, Nuala M Byrne, Grace K Munthali, James Chipeta, Ray Handema, Mofu Musonda, Andrew P Hills

**Affiliations:** 1 International Centre for Tropical Agriculture, Chitedze Agriculture Research Station, PO Box 158, Lilongwe, Malawi. (Affiliated with the National Institute for Scientific and Industrial Research during study implementation; 2 Institute of Health and Biomedical Innovation, Queensland University of Technology, 60 Musk Avenue, Kelvin Grove QLD 4001, Australia; 3 National Institute for Scientific and Industrial Research, PO Box 310158, Lusaka, Zambia; 4 University of Zambia, School of Medicine, Department of Paediatrics and Child Health, PO Box 50110, Lusaka, Zambia; 5 Tropical Diseases Research Centre (TDRC), PO Box 71769, Ndola, Zambia. (Affiliated with the National Institute for Scientific and Industrial Research during study implementation; 6 National Food and Nutrition Commission, Plot 5112, Lumumba Road PO Box 32669, Lusaka, Zambia; 7 Mater Mother's Hospital, Mater Medical Research Institute, Raymond Terrace, South Brisbane, QLD, Australia; 8 Griffith Health Institute, Griffith University, Parklands QLD, Australia

## Abstract

**Background:**

Zambia is a sub-Saharan country with one of the highest prevalence rates of HIV, currently estimated at 14%. Poor nutritional status due to both protein-energy and micronutrient malnutrition has worsened this situation. In an attempt to address this combined problem, the government has instigated a number of strategies, including the provision of antiretroviral (ARV) treatment coupled with the promotion of good nutrition. High-energy protein supplement (HEPS) is particularly promoted; however, the impact of this food supplement on the nutritional status of people living with HIV/AIDS (PLHA) beyond weight gain has not been assessed. Techniques for the assessment of nutritional status utilising objective measures of body composition are not commonly available in Zambia. The aim of this study is therefore to assess the impact of a food supplement on nutritional status using a comprehensive anthropometric protocol including measures of skinfold thickness and circumferences, plus the criterion deuterium dilution technique to assess total body water (TBW) and derive fat-free mass (FFM) and fat mass (FM).

**Methods/Design:**

This community-based controlled and longitudinal study aims to recruit 200 HIV-infected females commencing ARV treatment at two clinics in Lusaka, Zambia. Data will be collected at four time points: baseline, 4-month, 8-month and 12-month follow-up visits. Outcome measures to be assessed include body height and weight, body mass index (BMI), body composition, CD4, viral load and micronutrient status.

**Discussion:**

This protocol describes a study that will provide a longitudinal assessment of the impact of a food supplement on the nutritional status of HIV-infected females initiating ARVs using a range of anthropometric and body composition assessment techniques.

**Trial Registration:**

Pan African Clinical Trial Registry PACTR201108000303396.

## Background

Zambia is among the five countries in sub-Saharan Africa with the most serious HIV/AIDS epidemic [1]. In 2007, the HIV prevalence rate in adults was reported to be 14%, a slight but non-significant decrease from 16% in the 2001-2002 (Zambia Demographic Health Survey) [2]. Zambia also suffers from high rates of both macro- and micro-nutrient malnutrition. Indicators of nutritional status in children under five years of age show the prevalence of stunting, underweight and wasting at 45%, 15% and 5%, respectively. The prevalence of vitamin A deficiency and anaemia in the same age group is 54.1% and 52.9%, respectively [2-4]. Malnutrition compromises immunity to infectious diseases, which in turn aggravates malnutrition. The so-called malnutrition infection complex (MIC) is a well known phenomenon and the presence of HIV has made the situation worse. HIV/AIDS also drives household vulnerability to food insecurity, which contributes to households not having access to adequate food to meet the daily nutrient requirements for individuals to lead active and healthy lives [5].

Following the first diagnosed AIDS case in Zambia in 1984, the Government has put in place a number of strategies and interventions to fight the epidemic [6]. These interventions include preventive approaches to reduce new cases of HIV/AIDS infection and the provision of free ARV treatment [7]. To achieve the full benefit of ARV treatment, adequate nutrition is essential, therefore the government promotes good nutrition and in particular, HEPS is provided to PLHA, including those on ARV treatment. Commonly, HIV-infected individuals have reduced food intake due to the effect of the HIV infection compounded by secondary opportunistic infection. This has a marked effect on appetite leading to reduced food intake and consequently, to weight loss [8]. Weight loss in PLHA is dissimilar to that observed in severely malnourished individuals, as FFM rather than FM diminishes with the HIV infection. With the advent of ARV treatment, multiple additional nutritional changes which lead to obesity and the metabolic syndrome have been observed [9].

Typically, measurement of body weight is the nutritional assessment approach used to monitor health outcomes in resource-limited countries, with gain in absolute weight being the desired short-term outcome. Less common is the use of other anthropometric approaches such as circumference and skinfold thickness measurement, or objective body composition techniques. Bioelectrical impedance analysis (BIA) is a quick, non-invasive and relatively inexpensive body composition assessment technique [10,11] suitable for routine clinical use in the developing world. Skinfold anthropometry has been used to estimate body composition in HIV-infected persons using predictive equations however; as equations are population-specific, they need to be validated against a gold standard method before being used on another population [12].

This study aims to measure the impact of HEPS on the nutritional status and body composition of HIV-infected people who commence ARV treatment, over a 12-month period. In this study, body composition will be assessed using the stable isotope dilution technique to quantify TBW and subsequently FFM, along with body height, weight, BMI, skinfold and circumference measurements plus BIA.

Food intake and micronutrient status will also be assessed in the study population. This is based on the premise that the prevalence of macronutrient and micronutrient malnutrition, particularly vitamin A and iron, worsens with the HIV/AIDS epidemic. Further, as CD4 counts and viral load are important biomarkers of health status in this population, they will also be measured.

## Methods/Design

### Study design

This is a community-based controlled and longitudinal study. The study population will be comprised of HIV-infected females commencing ARV treatment at two Lusaka Urban clinics, Matero Reference Clinic (MRC) and Matero Main Clinic (MMC). The study participants will be allocated to either the experimental group and receive HEPS or control group and not receive HEPS. Participants will be randomly assigned to either group by the sister-in-charge at each clinic. Data will be collected at four time points across the 12-month study: baseline (0 months), 4-months, 8-months and 12-months follow-up. The study protocol has been approved by the University of Zambia Research Ethics Committee (approval ref 017-02-07).

### Recruitment

Participants will be recruited to the study from women who report to each clinic for care and support after being diagnosed as HIV-positive at a Voluntary and Counselling Treatment (VCT) centre in Lusaka. The clinics will collect blood samples for CD4 count, viral load and liver function tests from each study participant and subsequent appointment times to collect their results will be given. Blood samples collected in all government clinics in Lusaka are analyzed at the Centre for Infectious Diseases Research Laboratories in Lusaka. Upon receipt of results the sister-in-charge at each clinic will screen the results and identify those individuals considered to be eligible to commence ARV treatment based mainly on a CD4 count of 350 cells/μL or less.

Using a tracking system which consists of the participant's residential address and telephone number (or that of their spouse or guardian), eligible participants will be reminded of their clinic appointments. For those who do not have telephone contacts, a clinic staff member will visit them at their respective homes to inform them of their appointment and the need to report to the clinic and the Sister-in-Charge for ART. The sister will then invite each client one by one and explain the nature of the study. Once the client agrees to participate they will be asked to sign a consent form in duplicate or if they are unable to write, they will provide a thumb print. The Sister-in-Charge will sign as a witness. The original copy will be filed at the clinic while the duplicate copy will be stored by the Principal Investigator (PI) in a locked filing cabinet at the project office.

Once the client has consented to the study a project ID number will be assigned and the PI will meet with each individual to work through the information sheet in detail. During this period, each client is provided with another opportunity to clarify any issues or concerns before embarking on the study. Trained nursing and project staff will take routine measurements such as blood pressure and body temperature and also collect a urine sample for a pregnancy test. This will be followed by a full physical examination by a medical practitioner. Once the client has been certified as eligible to commence ARV treatment and meets all inclusion criteria she will be enrolled as a study participant.

Participants will be eligible if they are HIV-positive with CD4 count 350 cells/μL or less; are commencing ARV treatment; female aged 20-40 years; not mentally challenged and agree to participate in the study. Participants will be ineligible if clinically unstable or with other medical conditions (e.g., elevated creatinine), which will prevent them from commencing on ARV treatment, if they are pregnant or plan to fall pregnant during the study.

### Allocation to study groups

On the day of recruitment (baseline) after completing all body composition assessments and physical examination, the sister-in- charge will cut pieces of papers, equal to the number of participants enrolled that day. Half of the papers will bear the word 'Soya' while the other half will have 'No Soya' written on them. The papers will be folded several times to conceal the writing and then mixed. Each participant will be asked to pick one and read loud for the sister-in-charge to record the group assignment in the study registry book.

### Intervention/experimental group

A total of 120 kg of the food supplement will be given to each participant in the intervention or experimental group during the 12-month study period. To ensure a fresh supply of the food supplement and to minimise sharing or selling, the participants will be given 10 kg of the food supplement, enough for 30 days, and will be asked to collect 10 kg each month from the clinic. Each time the participant travels to collect the food supplement they will be given Zambian kwacha (ZMK) equivalent to approximately US$3 to cover the cost of transport from their home to the clinic. HEPS is a mixture of extruded maize meal and soya flour to which sugar and a vitamin-mineral mix have been added. Porridge is the common form in which HEPS is consumed. The participants will be given instructions on how to prepare the porridge from the HEPS and will be advised to consume it at least two times a day. In addition, they will receive further advice on the importance of good nutrition at the same time of taking ARV medication.

### Control group

Participants in the control group will not receive any food supplement but instead will receive a box of detergent paste and a packet of toilet soap once every 4 months at each scheduled follow-up visit. This group will also receive the same nutritional advice as the experimental group including medication.

### Training of project staff

The International Atomic Energy Agency (IAEA) will provide Fellowship training for two project team members, the PI and a senior laboratory technician. Training in body composition measurement techniques will be undertaken at Queensland University of Technology, Brisbane, Australia. The trained project staff will further refine their skills prior to training the field staff by completing pilot testing on 20 volunteer participants recruited from amongst the staff members at the research institution. This will assist in the preparation of a locally adapted training manual for subsequent use in the training of all field staff.

The use of BIA, skinfold thickness, circumference and isotope dilution techniques to assess nutritional status is not common in Zambia. Therefore, prior to implementing the field work a high level advocacy and awareness meeting for policy makers in public, private and international organisations dealing with nutrition and health will be organised to seek their support. Mixed presentations covering nutritional challenges, the consequences of HIV infection, interventions, traditional nutrition assessment tools and their limitations and state-of-the-art techniques will be made. Presenters will include renowned health practitioners, nutritionists, research scientists from various arms of government, and a technical expert from the IAEA.

### Recruitment and training of field staff

Six nurses, three from each of the two selected study site clinics, and two medical practitioners, one from each clinic, will be recruited and trained as field staff across two comprehensive training sessions. The aim of the first training session will be to introduce the anthropometric and body composition techniques and methodologies using both theory and practical demonstrations. In addition, a pre-test will be conducted at one of the clinics using 10 volunteer students from the Natural Resource Development College (NRDC) in Lusaka who are undertaking a food and nutrition diploma course. Ten students will be selected for the pre-test to provide the opportunity for a real world practical application of their theoretical work in class as this is the anticipated maximum number of participants likely to be recruited in a day.

The second training session will involve on-site training for the field staff at the respective clinics selected for the study. The training will be conducted a week prior to commencement of the recruitment of participants and baseline data collection. The aim of the on-site training will be to acquaint field staff with the sequence of events to be followed during data collection, have a final hands-on practice of the techniques and work out other logistical issues including space, laboratory and cafeteria facilities. A total of 50 female volunteer students from NRDC will be used as participants, 10 per day with three days training to be conducted at Matero Reference Clinic and two days at Matero Main Clinic. Students will be given appointment times to report to the clinics and will be refunded for transport expenses. However, measurements during the on-site training will be limited to body composition assessment. Physical examination by the medical practitioner, gravidex test, and collection of blood for assessing total blood count, CD4 counts, viral load and micronutrient analysis will not be undertaken. Similarly, tests relating to HIV/AIDS status and counselling will not form part of the pre-test.

## Outcome measures

### Anthropometry

Weight will be measured to the nearest 0.1 kg using a Seca digital portable scale (model 813 Seca gmbh & Co., Hamburg, Germany) with the participant wearing minimal clothing. Participants will be briefed about the importance of taking accurate weight measurements. HIV-infected people lose a considerable amount of weight before they seek medical care and clothes become very loose. To keep clothes in shape, many women wear several layers of clothing and if left unchecked, these could add considerably to the body weight. Each participant will be given a pre-weighed piece of cloth (two yard) to tie around their body at the time of measurement. All other clothes and accessories, with exception of underwear, will be removed.

Height will be measured to the nearest 0.1 cm using a free-standing Seca stadiometer (model 225 Seca gmbh & Co., Hamburg, Germany). A standard procedure for measuring height, without shoes and socks, will be followed. Height, if accurately measured, should remain constant across the duration of the study. However, it is common for African women to have elaborate hair styles that cannot be undone for the purpose of taking height measurements, therefore, where necessary, extra care will be taken in the estimation of height to account for such hair styles.

Circumference and skinfold thickness measurements will be done simultaneously since similar procedures are used to locate the sites. Three circumference measurements: mid-upper arm, abdominal at the level of the umbilicus, and hip will be taken using a Seca plastic measuring tape. Skinfold thickness will be measured at five sites: bicep, triceps, subscapular, iliac crest and medial calf using a Harpenden skinfold caliper (British Indicators Ltd., London, UK) with all measurements taken on the right side of the body. Standard procedures for the location of sites and taking measurements will be followed [13]. All measurements will be taken in triplicate. Circumference and skinfold thickness measures provide an indication of regional fat distribution. FM as a percentage of total body weight will be calculated from the sum of skinfold thickness measurements (biceps, triceps, subscapular and suprailiac sites = ∑4SF) and prediction equations [14].

### Bioelectrical impedance analysis

Impedance will be measured using a multi-frequency BIA (SFB7 model, ImpediMed Limited, Queensland, Australia) device according to the manufacturer's instructions. This will include ensuring that the battery is fully charged and the equipment is calibrated; participants will be asked not to exercise or take alcohol during the 24 hours before measurement. Participants will rest in a supine position for 5-10 min on a medical examination couch prior to measurement. A very mild current (50 ohms), imperceptible to the participant, passes through the body's water compartments. The SFB7 device takes measurements at 256 discrete frequencies, logarithmically spaced from 4 kHz to 1000 kHz. The software in the device enables the prediction of TBW and by extrapolation, FFM and FM. These prediction equations are population-specific hence there is a need to validate the results using the isotopic technique before the BIA device can be used routinely on HIV-infected people. Prior to analysis, each participant's height, weight, age and sex will be entered into the device.

### Total Body Water

A procedural requirement is that the deuterium oxide dose be administered to a fasted individual or at least two hours after taking a meal. Prior to administering the dose, a baseline saliva sample will be collected from each participant and used to determine the natural baseline enrichment of the isotope in the body. Participants will also be asked whether they have fasted and if not, the time they had their last meal will be noted. Participants will report to the clinic at 08.00 h and the deuterium dose will be administrated between 10.30 h and 11.00 h (as an additional precaution against participants having eaten recently). A pre-weighed dose of 30 g deuterium oxide will be administered orally to each participant regardless of body weight with the aid of a straw, followed by a rinse of the bottle (approximately 50 mL of mineral water) to ensure consumption of the full dose. Participants will be told not to eat, drink or void their bladder within one hour of ingesting the dose and 30 min before collecting the saliva sample. During the equilibration period, the consumption of drinks and emptying the bladder will be restricted. If this is necessary, volumes will be noted. However, due to the extended period of fasting, and period of assessment, participants will be provided with a standard lunch and drink and the volume of the drink will be noted. The first post-dose saliva sample will be collected at four hours after taking the dose and the second post-dose sample at five hours to ensure equilibration.

Saliva samples (4.5 mL) will be collected using a cotton wool ball and with the aid of a syringe and transferred into pre-labelled 5 mL vials and subsequently, pre-labelled zip-lock bags. Saliva samples will be transported in an insulated box cooled with freeze packs to the laboratory at NISIR and stored at -20°C for later analysis.

### Determination of TBW

Deuterium oxide enrichment of the saliva samples will be measured using a Fourier Transform Infrared (FTIR 8400S; Shimadzu, Vienna, Austria) spectrometer. This FTIR is available at the NISIR laboratories in Lusaka, Zambia. Prior to enrichment measurement of the saliva the FTIR will be calibrated using a 99.9 atom % deuterium oxide standard solution of known composition. The standard will be prepared according to the method described in the IAEA guidelines [15] by weighing deuterium oxide and diluting with local drinking water (bottled mineral water) to give a solution of about 1000 mg/kg by weight enrichment. This enrichment of the FTIR calibration standard will be verified using an Isotope Ratio Mass Spectrometer (IRMS) at a reference laboratory (Iso-Analytical Laboratory, UK). A range of enrichments will likely be encountered during analysis of saliva collected from the participants; the accuracy of deuterium oxide analysis over the range will therefore need to be checked by preparing a calibration curve. The 1000 mg/kg enrichment represents the maximum expected body water enrichment. The calibration procedure will involve preparation of deuterium oxide by gravimetric dilution (between 0 and 2000 mg/kg) and measuring their enrichment by the FTIR. Accuracy will be determined from the gradient and linear regression through the data of the calibration curve should be close to 1.

The FTIR will be set up according to IAEA guidelines [15-17]. Once ready to take measurements, the analytical precision of the FTIR will be checked before and after completing the measurements for the day by first taking measurements of local drinking water and the calibrator standard enrichment solution 3 times each and ensuring that the coefficient variation (CV) of less than 1% is achieved. Once the precision is ascertained the enrichment of the saliva samples for the participants will be measured. The spectrum of local drinking water will be obtained first as a background followed by measuring the spectrum of the standard in the range 2300-2800 cm^-1^. Thereafter the spectrum of the saliva will be taken by first obtaining the spectrum of the pre-dose saliva as background then followed by that of the post-dose samples (collected at 4 and 5 h). Prior to obtaining the spectra of the saliva, the samples will be thawed to room temperature and then centrifuged (Model 5702R, Eppendorf AG, Hamburg, Germany) at 3000 rpm for 10 minutes. Once the spectrum has been obtained it will be exported as a text file and further processed using the "isotope.exe" programme developed by the Medical Research Centre (MRC) [17]. This program enables the translation of the spectrum intensity into excess concentration of deuterium oxide in the saliva sample. The dilution space will be calculated from the concentration of deuterium oxide in the saliva using a standard formula. TBW (kg) will be calculated from the dilution space after correcting for non aqueous dilution of deuterium oxide. The dilution space is 1.04 times TBW. The FFM will be derived from TBW using a hydration coefficient, that is the fraction of FFM comprised of water and this is constant in adult, 0.732. Once FFM has been estimated, FM and percentage body fat will be calculated.

Quality control will be done in accordance with the IAEA guidelines [15]. The equation proposed by the IAEA for use as a quality control measure in TBW (kg) = 7.4 × height^3 ^(m^3^), where TBW from each post-dose sample is used with the height of each participant. Since this equation will not be derived from our population in particular, a range incorporating the 95% confidence interval for the above relationship (< 5.7 × height^3 ^or > 9.6 × height^3^) also proposed by the IAEA for assessment of accuracy, will be considered. The TBW samples falling outside this range will be excluded from the analysis. The variation in TBW values depends on both the degree of analytical precision and natural biological variation.

### Clinical assessment

Physical examinations will be undertaken by a medical practitioner and fasting blood sample collected. At baseline and at 12-month assessments, three vials of blood will be collected, for CD4 count, viral load and micronutrient analysis. At 4-month and 8-month follow-ups, all measures except micronutrient analysis will be undertaken. Part of the blood sample for CD4 count will be used to measure full blood count which will be determined at the clinic laboratory. Blood samples will be processed at the clinic laboratory to separate the plasma from the blood cell for CD4 and viral load and the serum for micronutrient analysis. The plasma for viral load and serum for micronutrient determination will immediately be stored at -20°C at the clinic until shipment, while the plasma for CD4 count will be stored at room temperature, packaged and couriered by FedEx to the TDRC in Ndola.

### Dietary intake study

A 3-day weighed food record coupled with a 24-h dietary recall technique will be used to determine the dietary pattern and the nutrient intake of the participants. Ten individuals with knowledge of nutrition will be recruited from various government institutions as "field staff". Training will include pre-testing on both 3-day weighed food records and 24-h dietary recall methodologies. Twenty participants will be recruited for the pre-test from another clinic in Lusaka offering ART services but not involved in the main study.

It is common practice for HIV-infected people to seek care and support from a health facility far from their residential catchment area. Therefore, it will be expected that many participants may be located in a wide range of residential locations of Lusaka including urban, peri-urban and rural areas. A well-designed house location plan will be required. Final updates on the residential address of each participant will be collected when they come for the 8-month follow-up visit. A participants' register to aid clustering based on their residential location will be prepared to assist in assigning participants within close proximity to the same field staff. In addition to telephone and house numbers, detailed directions to the participant's residence will be taken, including names of nearby landmarks such as schools, churches, and shops. It is also important to note the local names used to identify participants in their local communities. For example, it is common for women in Zambia to be called by their children or husband's names.

Visiting participants in their home might not be acceptable as some participants might not wish to disclose their HIV status to some or all members of their household. Participants will be assured that the all dietary intake data will be collected by non-medical personnel. This component of the study will be led by the National Food and Nutrition Commission which is well known as an institution responsible for promoting the nutrition agenda in the country which should help to minimize any potential stigma.

A total of 200 participants will be recruited for the study and a drop-out rate of 20% is anticipated. Accordingly, we anticipate that dietary intake data will be collected from approximately 160 participants. Each field staff member will be assigned 2 participants per day therefore we anticipate that data collection will take about two months to complete. Each participant will be contacted a week before data collection to remind them of the visit by field staff. Wherever possible, an understanding of level of literacy will be established beforehand. For participants who are unable to read and write, somebody in the household will need to be identified to record for them.

Field staff will train two participants per day, one in the morning and the second in the afternoon. This will be done on Mondays while Tuesday, Thursday and Saturday (or Sunday for Seventh Day Adventists) will be the 3 days during which the participants will be recording their food intake. On the second day (Wednesdays) the field staff will visit the participant's home to conduct a 24-h dietary recall. This will be done to assess the correct observance of the diet records, after keeping the record for one week day. The results of which will be immediately compared with those recorded for one day and will be the basis for reinforcing what will be discussed during the training. All forms and the food scales will be collected on Sundays, or Mondays for Seventh Day Adventists. Five supervisors will pay random visits to participant's homes to check on the accuracy and completeness of recording.

### Demographics

A questionnaire to assess demographic information will be completed by each participant on the day of recruitment and include questions on age, educational level, marital and socioeconomic status, and length of time they have known about their HIV status.

### Data management and entry

All measurements will be entered on forms specifically designed for the project. Due to the number of measurements, forms of different colours will be used for easy identification. Completed forms will be checked for completeness by the PI or any other NISIR project staff present at the clinic. Each clinic will maintain separate files with all files stored safely by the PI.

Clinical assessment data including micronutrient analysis and socio-demographic assessment will be entered using EPI-INFO software (Centres for Disease Control and Prevention, Atlanta GA) while BIA, skinfold thickness, circumference and TBW measurements will be entered in Excel data sheets. Dietary intake forms will be checked for completeness by the supervisors at NFNC. A spread sheet in Excel 2007 will be created in which raw data from the record forms consisting of the foods and amounts consumed will be entered. Data bases containing nutrients to be used in the analysis will be created using food composition tables for Zambia [18], Food and Agriculture Organisation [19] and Mali [20,21]. Recipe databases will be created using values from the 2009 NFNC study and the Uganda/HarvestPlus databases. Then the data base containing nutrients from the food composition tables will be merged with those containing the foods and amounts consumed to calculate the nutrient intake.

### Sample size calculation

The sample size calculations were based on detecting a 5% weight change between groups over the 12-month period. Assuming a 10% variance in the within-group body weight, 37 participants are needed in each group to detect this difference with an alpha of 0.05 and a power of 90%. If the attrition is 25% over the year, a minimum of 48 participants are required in each group. Assuming FFM is 35% of the weight gain [22], 44 participants are needed in each group to detect this difference with an alpha of 0.05 and a power of 90%. If the attrition is 25% over the year, a minimum of 58 participants are required in each group. It is uncertain what magnitude of change is expected in CD4 counts and viral load. For a moderate effect size (Cohen's d = 0.4) 66 participants are needed in each group to detect this difference with an alpha of 0.05 and a power of 90%. If the attrition is 25% over the year, a minimum of 88 participants are required in each group.

### Statistical Analysis plan

Statistical analyses will be performed using SAS software (SAS Institute, Cary, NC, USA). Descriptive statistics will be provided for the nutritional status, body composition, clinical and socio-demographic characteristics of the participants at baseline, 4, 8 and 12 months follow-up. At baseline, the characteristics of participants on food supplement and those without food supplement will be compared using t-test for continuous variables and Fisher's Exact Test for categorical variables. Repeated measures analysis of variance (RM-ANOVA) will be employed to compare the primary outcome variables (nutritional status and body composition) in participants with and without food supplements. Where confounding variables may need to be account for, adjustment will be made by covariance.

## Discussion

Few studies have used comprehensive anthropometric and criterion body composition assessment techniques to assess the impact of food supplements in Zambian HIV-infected women receiving ARV treatment and indeed elsewhere in sub-Saharan Africa. The outcome of this study will thus provide Zambia and the region with valuable information that will help guide the development of appropriate nutrition guidelines for PLHA, especially in the context of the risk for obesity and the metabolic syndrome in this population.

BIA is a rapid, inexpensive and non-invasive method to measure body composition and of particular interest for field and clinical studies in this setting. The ability to utilise criterion measures of nutritional status in these people will provide important information to inform more appropriate nutritional interventions and the provision of improved clinical care and support using validated approaches.

Despite the potential benefits of the BIA technique, a limitation is the inability to identify site-specific alterations in body fat, a particular issue in PLHA [23]. Skinfold thickness measurements provide information on regional and whole body composition and hence may be approach to consider visceral fat accumulation and fat loss from limbs, of clinical concern due to the relationship with insulin resistance and other serious metabolic disturbances [24].

One of the limitations of the proposed study is that only female participants will be included. Due to gender differences in body composition and HIV-associated wasting, results will not be readily applicable to HIV-infected males [25] and children.

## Competing interests

The authors declare that they have no competing interests.

## Authors' contributions

RM is the Principal Investigator, led the study design and manuscript writing. NB planned statistical data analysis. GKM contributed to the study design. JM designed and led the clinical assessment component of the study and provided advice on data management. RH contributed to the design of the whole study. MM provided input to the design of dietary intake of the study and led the component. AH provided technical input both on the study design and manuscript writing. All authors have read and approved the manuscript.

## Pre-publication history

The pre-publication history for this paper can be accessed here:

http://www.biomedcentral.com/1471-2458/11/714/prepub
